# Influence of early-life stress on hippocampal synaptic and network properties

**DOI:** 10.3389/fncir.2024.1509254

**Published:** 2024-12-19

**Authors:** Andrei Rozov, Anastasia Fedulina, Viktoriya Krut’, Rostislav Sokolov, Arina Sulimova, David Jappy

**Affiliations:** ^1^Federal Center of Brain Research and Neurotechnologies, Moscow, Russia; ^2^Institute of Neuroscience, Lobachevsky State University of Nizhny Novgorod, Nizhny Novgorod, Russia; ^3^Laboratory of Neurobiology, Kazan Federal University, Kazan, Russia

**Keywords:** early life stress, hippocampus, LTP, excitation, oscillations

## Abstract

According to the World Health Organization, the number of people suffering from depressive disorders worldwide is approaching 350 million. The consequences of depressive disorders include considerable worsening of the quality of life, which frequently leads to social isolation. One of the key factors which may cause depression in adulthood is early life stress, in particular, insufficient maternal care during infancy. Studies performed with children raised in orphanages have shown that long-term complete absence of maternal care (chronic early life stress) leads to vulnerability to emotional disorders, including depression, in adulthood. All of the above dictates the need for a deep understanding of the mechanisms of the pathogenicity of stress in neurogenesis. Therefore, the consequences of stress experienced in the early stages of development are actively studied in animal models. A large body of evidence has accumulated indicating stress-induced changes in gene expression and behavioral disorders in adulthood. However, the connection between the molecular biology of neurons and complex behavior runs through the synaptic connections linking these neurons into complex neural networks. In turn, coordinated activity in neuronal ensembles, achieved by a balance of synaptic excitation and inhibition, is the basis of complex behavior. Unfortunately, the effect of stress on synaptic interactions of neurons remains poorly understood.

## Introduction

The data accumulated to date suggest that both genetic and stress factors underlie the development of mental disorders. About a hundred years ago, Sigmund Freud suggested that early negative experiences lead to the creation of certain “organic elements” that serve as the basis for the formation of psychopathologies in adulthood. Accumulating evidence suggests that negative experiences early in life can cause changes in central nervous system functioning that lead to pathology in adulthood when faced with certain types of stress (Heim and Nemeroff, 2002). It is assumed that at the early stage of postnatal ontogenesis, the central nervous system has high plasticity, which decreases with maturation. This, on the one hand, leads to the formation of stable networks and the behavioral patterns associated with them, and, on the other hand, reduces the possibility of changing the already formed networks, which can determine the long-term preservation of pathological changes that occurred in the early stages of development. Nevertheless, the nature of changes in the central nervous system under the influence of early life stress (ELS) remains largely unclear. It should be noted that in recent decades, the influence of ELS on brain ontogenesis has been actively studied in model animals. The vast majority of these papers are devoted to studying the effect of stress on gene expression or behavior and memory. These aspects of the pathogenic effect of ELS are certainly of interest from both a scientific and a medical point of view. However, the functioning of the brain leading to specific behavioral responses is carried out through the coordinated activity of neural networks, which, in turn, depends entirely on synaptic interaction between neurons. Unfortunately, the aspects of the influence of early life stress on the plastic properties of excitatory and inhibitory synapses are currently poorly understood. In In this mini-review, we focus on the effects of ELS on synaptic plasticity and rhythmogenesis in the hippocampus, as this structure is involved in shaping the behavioral consequences of ELS.

## Consequences of early life stress at the synaptic level

There are three widely used protocols for ELS in the first postnatal weeks: repeated (5 to 21 times) separation from the mother for periods ranging from 15 min to 8 h (maternal separation [MS]), prolonged (usually for a day) weaning from the mother (maternal deprivation [MD]) and environmental stress without weaning from the mother (decrease in the amount of bedding material in the cage) (Chen and Baram, 2016; Daskalakis et al., 2015).

Despite the fact that the intensity of stress-driven changes depends on the type of protocol, the overall direction of alterations in the expression of the main stress response markers is the same in all three protocols: a significant increase in the level of corticosterone and a decrease in the level of glucose and leptin in the blood plasma, a decrease in the expression of corticotropin releasing factor, etc (Maniam et al., 2014). The protocols that involve prolonged separation from the mother, especially MD, also cause nutritional deficit during the critical development period (the first two weeks of life in rodents), nevertheless, all three protocols cause changes in the functioning of the hypothalamic-pituitary-adrenal axis. Studies of the delayed behavioral consequences of ELS in most cases reveal severe behavioral disturbances in adulthood, including worse performance in the novel object recognition test (Llorente et al., 2011; Solas et al., 2010; Suri et al., 2013).

The generally accepted cellular equivalent of learning and memory is long-term (hours, days) changes in the efficiency of synaptic transmission in response to a specific pattern of neural network activity–a phenomenon known as long-term synaptic plasticity. There is evidence suggesting that long-term potentiation (LTP) may be significantly affected by ELS (Table 1). In a few studies is has been shown that in adult animals exposed to ELS, LTP at Schaffer collateral to CA1 pyramidal cell synapses can be selectively suppressed. However, different groups obtained rather contradictive results. For instance, Lesuis et al. (2019) reported that ELS induced by housing female mice and their offspring in conditions of limited nest and bedding material from day 2 to day 9 after birth resulted in decreased expression of the CluN2B subunit of NMDA receptors and occlusion of LTP at the Schaffer collateral input to CA1 pyramidal cells in adult males. However, a recent study reports that NMDAR function was selectively increased in adult female mice that underwent ELS (Wilkinson et al., 2024). The same group also found that ELS had no effect on LTP in either sex. Moreover, the synaptic plasticity modulation depends on the ELS protocol, the species and strain of experimental animals, age, and sex. One study reported an increase in LTP at CA1 pyramidal cell synapses in rats in response to MD (Derks et al., 2016), in males the increase in LTP was significant in young animals (P22-24), whereas in females LTP was increased only in adult mice (P85-95). MS generally results in decreased Schaffer collateral LTP (Herpfer et al., 2012; Sousa et al., 2014). However, Ryan et al. (2009) published data suggesting that MS caused both increased AMPAR and NMDAR expression and increased LTP in rat CA1 pyramidal cells. Interestingly, even longer periods of MS (P2–20, 4 h daily) do not affect LTP at Schaffer collateral input to CA1 neurons, but caused significant LTP reduction at mossy fiber input to CA3 pyramidal cells (Shin et al., 2016).

**TABLE 1:** Differential effects of ELS on LTP at distinct hippocampal synapses.

Species/(gender)	ELS-protocol	ELS-period	Tested input/(age)	Recording mode	LTP protocol	Results	AMPAR/NMDAR	References
Mice (M)	Environmental stress	P2-9	SC-CA1 (6 months)	fEPSP	HFS	LTP 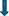	CluN2B 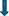	Lesuis et al., 2019
Rats (M & F)	MS	Pl-14	SC-CA1 TA-CA1 P71-111	WC patch clamp	TBS	No effect	NMDAR 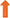	Wilkinson et al., 2024
Rats (M & F)	MS	P2-14	SC->CA1 (70 weeks)	fEPSP	HFS	LTP 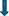	Not tested	Sousa et al., 2014
Rats (M & F)	MS	P2-14	SC-CA1 (adult)	fEPSP *in vivo*	HFS	LTP 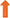	GluNl & GluA2/3 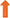	Ryan et al., 2009
Mice (M & F)	MS	Pl-14	SC-CA1 (P15; P70)	WC patch clamp	TBS	P15: no effect P70: LTP 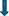	Not tested	Herpfer et al., 2012
Mice (M)	MS	P2-20	SC-CA1 MF-CA3 (P35-55)	fEPSP	TBS	SC-CAl: LTP no effect MF-CA3: LTP 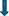	Not tested	Shin et al., 2016
Rats (M & F)	MD	P3	SC-CA1 (P22-24; P85-95)	fEPSP	HFS	M P22-24: LTP 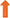 F P85-95: LTP 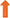	Not tested	Derks et al., 2016
Rats (M)	MS	P2-9	PP-DG (P28-30)	fEPSP *in vivo*	TET	LTP 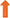	Not tested	Kehoe et al., 1995
Rats (M & F)	MS	P2-9	PP-DG (P28-30)	fEPSP *in vivo*	TET	M:LTP 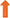 F: LTP no effect	Not tested	Bronzino et al., 1996
Rats (M & F)	MS	P2-9	PP-DG (P28-30)	fEPSP *In vivo*	TET	M:LTP 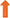 F: LTP 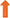	Not tested	Kehoe and Bronzino, 1999
Rats (M)	MD	P3	PP-DG (13 weeks)	fEPSPs	TBS	M; LTP no effect	Not tested	Oomen et al., 2010
Rats (F)	MD	P3	PP-DG (13 weeks)	fEPSPs	TBS	F; LTP no effect	Not tested	Oomen et al., 2010
Rats (M)	MS	P2-9	BLA-DG (3-4 months)	fEPSP *in vivo*	TBS	M:LTP 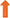	Not tested	Blaise et al., 2008

SC, Schaffer collaterals; MF, mossy fibers; PP, perforant path; BLA, basolateral amygdala; TET, tetanization; TBS, theta bust stimulation; HFS, high frequency stimulation; M, males; F, females; arrow up, enhanced LTP or GluR function; arrow down, reduced LTP or GluR function.

In addition to the changes of the local hippocampal excitatory synapses, ELS affects the properties of the cortical projections to the dentate gyrus (DG). However, while MS results in increased LTP at perforant pathway input to DG granule cells (Blaise et al., 2008; Bronzino et al., 1996; Kehoe and Bronzino, 1999; Kehoe et al., 1995), MD had no effect on LTP (Oomen et al., 2010; Oomen et al., 2011). Despite the fact that the primary description of the effects of ELS on LTP at hippocampal excitatory synapses was carried out in the above-mentioned studies, the lack of a systematic approach to this problem, covering the three main types of local excitatory synapses, does not allow us to form a complete picture of the long-term influence of ELS on synaptic plasticity at hippocampal glutamatergic synapses (Table 1). Moreover, since the molecular determinants of long-term potentiation in the hippocampus change during ontogenesis (Jensen et al., 2003), an important issue is to study the developmental profile of the effect of ELS on early forms of synaptic plasticity.

Despite the fact that there is no need to prove the primacy of excitation in maintaining communication between neurons, the normal functioning of the neural network is equally critically dependent on the synergistic interaction of inhibitory and excitatory neurons. Therefore, any stress-induced change in the inhibition pattern will affect the functionality of the hippocampus. In adult animals, stress affects the GABAergic networks at several levels, ranging from effects on the functioning of individual GABAergic synapses (Holm et al., 2011; MacKenzie and Maguire, 2015; Nasretdinov et al., 2023) to a decrease in the number of cells in certain subpopulations of GABAergic interneurons (Czéh et al., 2015). Although, the consequences of acute stress in adult animals are mostly reversible, chronic stress leads to the selective death of interneurons expressing parvalbumin (PV), neuropeptide Y, somatostatin and calretinin (Albrecht et al., 2021; Hu et al., 2010), increasing innervation provided by cholecystokinin (CCK) positive cells (Brito et al., 2024) and does not affect the number of neurons in subpopulations expressing calbindin or somatostatin. Among the listed subpopulations of interneurons, the most interesting are the two groups of interneurons that provide perisomatic inhibition of excitatory neurons in all fields of the hippocampus, namely, PV- and CCK-positive interneurons.

## Perisomatic inhibition and stress

GABAergic neurons fall into two main categories: cells that primarily innervate the perisomatic membranes or those that innervate neuronal dendrites. The perisomatic region is defined as the plasma membrane domain that includes the proximal dendrites, the cell body, and the axon initial segment. Perisomatic inhibitory neurons can more effectively control the generation of action potentials and thereby determine the probability and spiking frequency of the target cells. Perisomatic inhibition in the hippocampus is carried out by two types of interneurons: CCK- and PV-positive. Despite the fact that these interneurons receive excitatory inputs from identical hippocampal fields and innervate the same subpopulations of excitatory neurons, as mentioned above, the physiological role of these interneurons is significantly different. The role of PV-expressing basket interneurons in the generation of gamma oscillations has been widely demonstrated in both wild-type animals and numerous lines of genetically modified mice (Fuchs et al., 2001; Fuchs et al., 2007; Hormuzdi et al., 2001; Wulff et al., 2009). The function of these interneurons in generating the gamma rhythm (30–100 Hz) is traditionally determined by their participation in feedforward and feedback inhibition, as well as the ability of these interneurons to synchronize activity. In turn, the contribution of feedforward and feedback inhibition depends on the strength of the excitatory inputs to the interneurons, probabilities of GABA release and synchronization via gap junctions within the network of PV-positive neurons. A decrease in the efficiency of any of the above aspects of the functioning of the PV-expressing interneuron network leads to a significant reduction in the power of gamma oscillations.

Returning to the problem of the influence of ELS on perisomatic inhibition, it should be noted that a number of questions remain open: (1) How does ELS, for example, through the mechanisms of long-term potentiation and depression, affect the inputs and outputs of PV-positive cells? (2) How does stress-induced selective death of PV-positive interneurons affect the electrical connectivity and synchronization of interneurons? (3) Is it possible to compensate for the drop in perisomatic inhibition caused by the death of PV-positive neurons by additional recruitment of CCK-positive interneurons into network activity?

The proposed involvement of the CCK-positive perisomatic interneuron subpopulation in the formation of the post-stress response follows from *in vivo* experiments demonstrating stress-induced changes in the hippocampal theta rhythm (Murthy et al., 2019). The functioning of CCK-positive perisomatic synapses is controlled by the endocannabinoid signaling system through the activation of presynaptic CB1 receptors. Numerous studies show changes in endocannabinoid tone and expression level of cannabinoid receptors in response to various types of stress (Evanson et al., 2010; Hill et al., 2009; Wang et al., 2010), including ELS (Marco et al., 2014; Suárez et al., 2009; Viveros et al., 2009). Moreover, these changes often have a pronounced gender dependence (Reich et al., 2009; Suárez et al., 2009). MD was found to significantly reduce the density of CB1 receptors in the CA1 and CA3 fields of the hippocampus in adult animals (Suárez et al., 2009). Another article states that at the mRNA level, expression of CB1 receptors persists after MD, but the expression of genes responsible for the metabolism (synthesis and degradation) of endocannabinoids changes (Marco et al., 2014). In general, there is insufficient data in the current literature on the stress-driven modulation of various components of the endocannabinoid cascade, which includes the metabolism of endocannabinoids and the functionality of CB1 receptors. Despite the significance of the above studies, the lack of proven specificity of stress-induced changes in endocannabinoid sensitivity at the level of identified synapses leaves a wide window for speculation. For example, cannabinoid receptors expressed at excitatory synapses in the hippocampus may also be targets for stress-induced increases in endocannabinoid tone. The possible effect of ELS through the endocannabinoid signaling system on the formation and functioning of inhibitory feedforward and feedback loops, that include CCK-positive interneurons, has not been studied at all.

## Influence of early life stress on hippocampal rhythmogenesis

A number of *in vitro*, *in vivo* and *in silico* studies indicate an important role of PV-positive interneurons in hippocampal rhythmogenesis (Buzsáki and Wang, 2012; Chatzikalymniou et al., 2021; Freund and Buzsáki, 1996; Klausberger and Somogyi, 2008; Li et al., 2017). Even “small disruptions” in the proper functioning of the PV-positive cell network, reduction of excitatory drive onto these cells or occlusion of gap-junction coupling, usually lead to noticeable changes in gamma oscillations (Buhl et al., 2003; Fuchs et al., 2001; Fuchs et al., 2007; Hormuzdi et al., 2001). Thus, the partial loss of several interneuron types caused by ELS, including PV-positive basket cells (Murthy et al., 2019), should have consequences for network oscillations, yet to date there are only a few studies that have attempted to characterize the effects of ELS on hippocampal rhythms. Especially, taking into account the well documented behavioral consequences of ELS, one may expect to see a serious alteration in the theta rhythm (4–8 Hz), a neural oscillation in the brain that underlies various aspects of cognition and behavior, including learning, memory, and spatial navigation (Seager et al., 2002). Murthy et al. (2019) showed that in adult mice subjected to MS, theta power as well as alpha/beta power increased compared to controls when the animals were placed in a new environment. They also show that in ventral hippocampus theta-gamma coupling is inherently enhanced in stressed mice compared to controls (Murthy et al., 2019). MS in rats has also been shown to result in an increase in total sleep time and, in particular, an increase in time in the REM stage. These MS-induced changes in sleep pattern have been associated with an increase in theta oscillations in the hippocampus, amygdala, and cortical circuits (Sampath et al., 2014).

However, there is evidence that in rats, MS did not affect the power of theta and beta oscillations in the hippocampus, but reduced the power of theta and beta oscillations in the prefrontal cortex only in males exposed to stress (Reincke and Hanganu-Opatz, 0000). Additionally, the same study showed that MS significantly reduced theta-gamma cross-frequency coupling between the hippocampus and prefrontal cortex, selectively in males. It should be noted that the latter report presented data obtained in anesthetized animals, whereas Murthy et al. (2019) observed increased hippocampal theta oscillations in freely moving animals exposed to a novel environment.

Very little is known about ELS effects on gamma oscillations. Dricks (2016) analyzed the effect of MS on the developmental profile of *in vitro* measured gamma oscillations recorded in slices of dorsal hippocampus from young rats (P15-21), they found in control animals gamma power had highly significant correlations with both age and weight, while gamma in stressed rats was not correlated with either factor.

While the influence of ELS on theta and gamma rhythms has been the focus of recent research, the influence of stress during the critical period of CNS development and maturation on sharp-wave ripples (SPW-R) has been completely overlooked. SPW-Rs are oscillatory events produced by extremely synchronized neuronal activity of neurons in the hippocampal formation which occur spontaneously in waking states or during non-rapid eye movement sleep (Buzsáki et al., 1992). There is evidence pointing to the vital role of PV-positive basket cells in the generation of this type of network activity. It has been shown that in adult mice acute and chronic stress results in significant enhancement of both amplitude and duration of sharp-wave ripple oscillations. Moreover, in stressed animals the firing rate of CA1 pyramidal cells was selectively increased specifically during SPW-R, resulting in an increase in neuronal synchrony (Tomar et al., 2021). In adult mice social defeat stress caused selective increased frequency of SPW-Rs in the ventral hippocampus, but not in the dorsal hippocampus (Shiozaki et al., 2023). Coming back to ELS, it has been shown that MS changes the spiking pattern of principal cells in multiple brain regions shifting the balance between regular spiking and bursting activity toward the latter. ELS-induced enchased burst firing was observed in thalamic reticular nucleus and hippocampal pyramidal cells (Ali et al., 2013). Given the above-mentioned change in neuronal firing activity caused by ELS, one might also expect to see changes in SPW-R oscillations.

## Discussion

Animal models of stress at an early age in rodents, which were first used more than 50 years ago (Levine, 1957), have become widespread. A cross-search of the PubMed database using the terms “early stress” and “behavior,” “gene expression,” “synaptic plasticity” or “synaptic transmission” reveals that the focus is on the genetic, biochemical and behavioral consequences of ELS. However, the primary function of neurons is to receive, process and transmit information encoded in the form of postsynaptic responses, action potentials, firing rates and stability of neurotransmitter release at presynaptic terminals. Unfortunately, the effects of ELS on these parameters have received the least attention from the scientific community.

Existing studies of ELS effects on synaptic plasticity often report contradictive results that can be grouped as following:

(!) ELS results in suppression of LTP and/or decreased NMDAR function (Herpfer et al., 2012; Lesuis et al., 2019; Sousa et al., 2014).
(!!) ELS has no effect on LTP (Oomen et al., 2010; Oomen et al., 2011; Wilkinson et al., 2024).
(!!!) ELS leads to LTP enhancement and/or to an increase of NMDAR function (Blaise et al., 2008; Bronzino et al., 1996; Kehoe and Bronzino, 1999; Kehoe et al., 1995).

**FIGURE 1:**
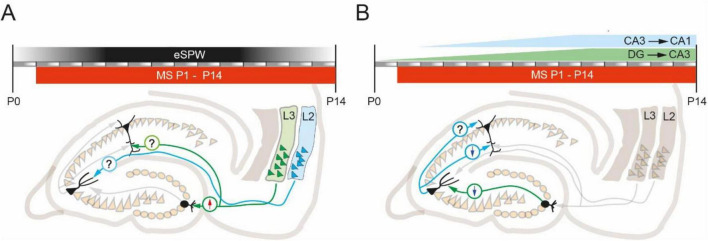
The commonly used window of MS induction protocol overlaps with the major crucial developmental events that might be affected by ELS. **(A)** ELS modulation of excitatory long-range projections from the entorhinal cortex to hippocampal targets. In the first two postnatal weeks, eSPWs provide sensory-driven inputs necessary for the proper development of projections from EC. MS results in increased LTP (arrow up) at EC-DG-granule cell synapses. Nothing is known about the effect of ELS (MS) on plastic properties at EC-CA3 and EC-CA1 synapses. **(B)** ELS modulation of intrahippocampal excitatory connections. The time courses of mossy fiber (green) and Schaffer collateral (blue) synaptogenesis overlap with the typical MS induction window. Most published studies show that MS reduces LTP (arrow down) at CA3-CA1 synapses located on apical dendrites and at DG-CA3 synapses. Nothing is known about the effect of ELS (MS) on plastic properties at CA3-CA1 synapses located on basal dendrites.

The apparent discrepancy between the data presented by different groups may be the result of differences in the protocols used to induce ELS. For example, MS and MD have different effects on glutamate receptor expression profiles and synaptic plasticity. Moreover, similar stress induction protocols vary across laboratories. In particular, the day after birth on which pups are separated from their mothers, as well as the duration of separation and the number of days the animals are exposed to the stressor, vary across studies. At present it is not possible to narrow the window in which ELS has the strongest impact on plastic properties at excitatory synapses in adult animals. However, there is evidence that the development of the intrinsic circuits of the entorhinal-hippocampal network requires sensory inputs transmitted by upper layer entorhinal stellate cells (Donato et al., 2017). This input to DG granule cells, CA1 and CA3 pyramidal neurons comes in the form of early sharp-waves (eSPW) throughout the entire critical period (P0-14). Electrophysiologically eSPWs are very similar to adult SPW-Rs, besides the absence of high frequency ripple oscillations. While adult SPW-Rs are spontaneous and self-generated in the hippocampal circuit events, generation of eSPWs involves inputs from the entorhinal cortex, which are activated during myoclonic movements. The peak of eSPW activity is around P5-7. Thus, the main “damaging action” of ELS might also occur during this period. However, temporoammonic inputs to CA1 that are recruited by eSPWs mature earlier than the Schaffer collateral inputs from CA3 (Cossart and Khazipov, 2022), so ELS may differentially affect individual connections at different time points depending on their place in the hippocampal hierarchy (Figure 1).

In addition to the high variability of stress protocols, the reported effects of ELS on synaptic plasticity have been tested using different approaches to induce and record LTP. Assuming that ELS can alter the expression profile of NMDARs, the best LTP induction protocol should be the most dependent on CA^2 +^ influx through these channels. Probably the best choice is a pairing of postsynaptic depolarization with low-frequency presynaptic stimulation. The use of theta-burst or tetanic stimulation may recruit dendritic voltage-gated calcium channels, which will counteract the deficit in NMDAR-mediated CA^2 +^ entry. It is important to assess ELS-dependent modulation of plastic properties on inputs originating from the same source but converging on functionally distinct cellular compartments. For example, in 25% of hippocampal pyramidal cells, the axon originates from one of the basal dendrites, which places inputs to the axon-carrying dendrite outside the control of perisomatic inhibition (Hodapp et al., 2022). Moreover, at CA3-CA1 pyramidal cell synapses, LTP on apical dendrites can be modulated by NO, whereas potentiation on the basal dendrite is NO-insensitive (Shiozaki et al., 2023). Shank 2 proteins differentially affect both AMPAR expression and LTP magnitude in individual lamellae of hippocampal CA1 dendrites (Eltokhi et al., 2021). Thus, to search for synaptic correlates of ELS-impaired behavior and/or oscillatory activity, we need more detailed, input-specific information on the effects of ELS on plasticity.

Another level of complexity in ELS research arises from the fact that stress during early postnatal development also alters astrocyte function (Çalışkan et al., 2020). Given that astrocytes interact with neurons in several ways, influencing neurotransmitter uptake and metabolism, providing gliotransmitters, and providing energy to neurons in local circuits, their involvement in ELS modulation of synaptic plasticity and rhythmogenesis also requires closer examination. The same is true when comparing studies on the effects of ELS on oscillatory activity. The variability of the ELS protocols and recording approaches used in the rather limited number of published studies does not allow us to draw firm conclusions about the impact of ELS on adult rhythmogenesis. At present, we can assume that ELS affects theta rhythms in various brain regions and probably the theta-gamma coupling.

Theta and SPW-R oscillations are necessary for learning and related to different behavioral patterns. The theta rhythm usually dominates when animals are actively engaged in acquiring information about their surroundings. There is the hypothesis that theta oscillations are involved in special navigation by controlling learned hexagonal spatial firing patterns of entorhinal grid cells. Experimental reduction of the theta rhythm by inactivating the septal projections causes a reduction in the hexagonal spatial firing patterns of grid cells (Brandon et al., 2011; Koenig et al., 2011). The role of theta oscillations in learning was also shown in experiments with hippocampus-dependent eyeblink conditioning. Administering training trials in the explicit presence or absence of hippocampal theta oscillations can either accelerate or retard the learning rate, respectively.

SPW-R, in contrast to theta oscillations, are thought to be mostly involved in the consolidation of recently processed information (Buzsáki, 2015). Suppression of hippocampal SPW-R during a critical period after training on a spatial learning task severely retards learning (Girardeau et al., 2009). Thus, ELS-dependent reductions in theta activity and SPW-R (the latter, if found) may result in learning deficits during both information acquisition and memory consolidation.

It seems that the effects of ELS on oscillations are more pronounced when animals are exposed to another stressor during recording or during complex task-driven behavior. The latter limits further investigation of the effects of ELS on brain rhythmogenesis to *in vivo* studies as a primary approach. Finally, it would be more beneficial if electrophysiological studies of ELS effects were to combine characterization of synaptic properties in slices with assessment of possible changes in oscillatory activity *in vivo.*
